# Correction: Forecasting the impact of population ageing on tuberculosis incidence

**DOI:** 10.1371/journal.pone.0224673

**Published:** 2019-10-28

**Authors:** The PLOS ONE Staff

## Notice of Republication

An incomplete, earlier version of this article was published in error. The Materials and methods incorrectly included URLs that did not relate to the article content. The publisher apologizes for this error. This article was republished on October 16, 2019 to correct for this error. Please download the article again to view the correct version.

## Supporting information

S1 File Originally published, uncorrected article. (PDF)

S2 File Republished, corrected article. (PDF)
