# Supplementary material for: Forecasting the impact of population ageing on tuberculosis incidence
Source: PLoS One. 2019 Sep 24;14(9):e0222937. doi: 10.1371/journal.pone.0222937 (PMC6759178; doi:10.1371/journal.pone.0222937)
Supplement: S1 Appendix — (PDF) [file pone.0222937.s001.pdf]

Specification of the Lee-Carter approach

1.1 Notations and abbreviations

$y_{age,year}$: number of events of *age* in *year*

$n_{age,year}$: population size of *age* in *year*

α_{age}: age-specific effect of *age*

β_{age}: age-period adjustment term of *age*

κ_{year}: period effect at *year*

$E(.)$: expectation function

$sign(.)$: sign function of a value, 1 for positive and -1 for negative.

$\ell(.)$: log-likelihood function

$ARIMA(p,d,q)$: Autoregressive integrated moving average with p number of time lags of the autoregressive term, d order of differencing term, and q number of time lags of the moving-average term.

1.2 Model structure and assumptions

Lee-Carter model [1] was defined as

$$log(E(y_{year,age})) = \alpha_{age} + \beta_{age}\kappa_{year} + log(n_{year,age})$$

Two constraints are needed for ensuring identifiability:

$$\sum_{year} \kappa_{year} = 0$$
$$\sum_{age} \beta_{age} = 1$$

In implementation, we used a Poisson-regression-based approach [2] for the Lee-Carter model

$$y_{year,age} \sim Poisson(\mu_{year,age})$$
$$\mu_{year,age} = n_{year,age} exp(\alpha_{age} + \beta_{age}\kappa_{year})$$

Thus, the log-likelihood function is

$$\ell(\alpha_{age}, \beta_{age}, \kappa_{year}|y_{year,age}, n_{year,age}) = -\mu_{year,age} + y_{year,age} log(\mu_{year,age}) - log(y_{year,age}!) \quad (1.1)$$

$$= y_{year,age}(\alpha_{age} + \beta_{age}\kappa_{year}) - n_{year,age} exp(\alpha_{age} + \beta_{age}\kappa_{year}) + constant \quad (1.2)$$

1.2.1 Comparator models

Two reduced models were considered as comparators:

Age-Period model assumed β_{age} are the same for all age groups, so the model reduced to

$$y_{year,age} \sim Poisson(\mu_{year,age})$$
$$\mu_{year,age} = n_{year,age} exp(\alpha_{age} + \kappa_{year})$$

Age-Trend model assumed β_{age} are the same for all age groups and a linear period effect, so the model reduced to

$$y_{year,age} \sim Poisson(\mu_{year,age})$$
$$\mu_{year,age} = n_{year,age} exp(\alpha_{age} + year \times \kappa)$$

1.3 Model fitting

The maximum likelihood estimation of this model is to solve

$$\underset{\alpha_{age},\beta_{age},\kappa_{year}}{\text{argmax}} \ \ell(\alpha_{age}, \beta_{age}, \kappa_{year} | y_{year,age}, n_{year,age})$$

subject to

$$\sum_{year} \kappa_{year} = 0$$
$$\sum_{age} \beta_{age} = 1$$

We employed Newton methods as described in Brouhns et al. [2] to this task. Our implementation used *StMoMo::fit* function from package StMoMo [3].

1.4 Modelling and forecasting

For period effects, κ_{year}, we employed the Box-Jenkins method, which uses autocorrelation function (ACF), partial autocorrelation function (PACF), and extended ACF if necessary to specified a time-series model of κ_{year}. This specification and modelling were implemented using functions,*TSA::acf*, *TSA::pacf*, and *TSA::eacf* from package *TSA* [4].

1.5 Bootstrap

The bootstrap simulation employed the semi-parametric bootstrap by Renshaw and Haberman [5]. Our implementation used *StMoMo::simulation* function from package StMoMo [3]. In general, we generated 10,000 simulations for each presented result.

1.6 Measurements of goodness of fit

Since the likelihood-based LCM we applied is a special case of the ordinary Poisson regression [6], the measurements of goodness of fit of Poisson regression can be directly applied.

Akaike information criterion (AIC): by definition,

$$2k - 2\hat{\ell}(\hat{\alpha}_{age}, \hat{\beta}_{age}, \hat{\kappa}_{year} | y_{year,age}, n_{year,age})$$

, where $\hat{\ell}(.)$ is the log-likelihood function given estimated parameters, k is the number of parameters, which equals *the sum of the numbers of* $\hat{\alpha}_{age}$, $\hat{\beta}_{age}$, $\hat{\kappa}_{year}$ *minus two constraints*.

Bayesian information criterion (BIC): by definition,

$$log(o)k - 2\hat{\ell}(\hat{\alpha}_{age}, \hat{\beta}_{age}, \hat{\kappa}_{year} | y_{year,age}, n_{year,age})$$

, where k is the number of parameters as above and o is number of observations.

Deviance residuals: We used the deviance residuals defined in Colin Cameron and Trivedi [6] to assess the goodness of fit.

$$sign(y_{age,year} - E(y_{age,year}))\sqrt{2\left[y_{age,year} log\frac{y_{age,year}}{E(y_{age,year})} - (y_{age,year} - E(y_{age,year}))\right]}$$

Bibliography

[1] Lee RD, Carter LR. Modeling and Forecasting U. S. Mortality. J Am Stat Assoc. 1992;87(419):659–671.

[2] Brouhns N, Denuit M, Vermunt JK. A Poisson log-bilinear regression approach to the construction of projected lifetables. Insur Math Econ. 2002 Dec;31(3):373–393.

[3] Andres V, Millossovich P, Vladimir K. StMoMo: Stochastic Mortality Modeling in R. J Stat Softw. 2018;84(3):1–38.

[4] Chan KS, Ripley B. TSA: Time Series Analysis; 2018.

[5] Renshaw AE, Haberman S. On simulation-based approaches to risk measurement in mortality with specific reference to Poisson Lee–Carter modelling; 2008.

[6] Colin Cameron A, Trivedi PK. Regression Analysis of Count Data. Cambridge University Press; 2013.
