# Supplementary material for: Forecasting the impact of population ageing on tuberculosis incidence
Source: PLoS One. 2019 Sep 24;14(9):e0222937. doi: 10.1371/journal.pone.0222937 (PMC6759178; doi:10.1371/journal.pone.0222937)
Supplement: S2 Appendix — (PDF) [file pone.0222937.s002.pdf]

Synthetic population methods

2.1 Background

The aim of this document is to describe the construction of a synthetic population of Taiwanese nationals as well as the basic population features based on the simulation of the model. The synthetic population will be used as an input of the TB modelling.

The National Development Council, Taiwan (NDC), developed a synthetic population for policy-making purposes and release a population projection report annually [1]. However, the model did not capture the stochasticity in its subprocesses and we required the lifespan between 0 and 100. Based on the assumptions and model choices in the NDC model, we constructed a stochastic synthetic population model for use in the TB incidence modelling.

The main features of our synthetic population were as follows.

- Modelling lifespan from 0 to 100 (assumed deterministic deaths when reaching aged 101).

- Being capable of generating stochastic population forecasts.

2.2 Notations and abbreviations

Data and demographic features

$year$: calender year

age: singe-year age

agp: five-year age group

sex: biological sex at birth

$PF_{year,age,sex}$: population size on 1st January of $year$

$PM_{year,age,sex}$: population size on 1st July of $year$

$PE_{year,age,sex}$: population size on 31st December of $year$

$D_{year,age,sex}$: deaths during $year$

$d_{year,age,sex}$: death rate at $year$

$B_{year,sex}$: number of newborns during $year$

$F_{year,agp,sex}$: number of newborns of sex during $year$, given agp of mothers

$f_{year,agp,sex}$: fertality rate of sex at $year$ given agp of mothers

$M_{year,age,sex}$: number of net migrations during $year$

$m_{year,age,sex}$: net migration rate at $year$

Lee-Carter components:

$\alpha_{age,sex}$: age-specific effect of (age, sex)

$\beta_{age,sex}$: age-period adjustment term of (age, sex)

$\kappa_{year,sex}$: period effect at $year$

$E(.)$: the expectation of a random variable

$ARIMA(p,d,q)$: Autoregressive integrated moving average with time lag p for the autoregressive term, order d for the differencing term, and lag q for the moving-average term.

2.3 Data

The demographic data were obtained from the Department of Statistics, the Ministry of the Interior, Taiwan. They were released publicly and available on the internet. All the training data in this article were published by the Taiwan officials and free access on the internet; the usage is licensed by the Open Government Data License: [https://data.gov.tw/license].

End-year population estimates. The estimated population size on 31st December of a year. These data were from 2004 to 2017 by sex, single-year age. We take them as the equivalent of the start-year population estimates from 2005 to 2018.

Death counts. The number of deaths on registration of a year. These data were from 2005 to 2017 by sex and single-year age.

Fertility counts. The number of births on registration of a year. These data were from 2005 to 2017 by sex of newborns and five-year-age groups (15-19, ..., 45-49) of mothers. We considered the childbearing ages of females from 15 to 49.

2.4 Modelling and forecasting

The synthetic population considers birth, death, ageing, and migration processes. As the NDC model, we used cohort-component methods [2] to combine them and drive the population dynamics. The essential model is:

$$\begin{aligned} PE_{year,0,sex} &= B_{year,sex} + M_{year,0,sex} && age = 0 \\ PE_{year,age,sex} &= PF_{year,age,sex} - D_{year,age,sex} + M_{year,age,sex} && age \in [1, 100] \\ PF_{year,0,sex} &= 0 && age = 0 \\ PF_{year,age,sex} &= PE_{year-1,age-1,sex} && age \in [1, 100] \end{aligned}$$

For all submodels, we generated 10,000 bootstrap simulations for considering stochasticity with the time range from 2000 to 2017 for fitting and from 2018 to 2035 for forecasting.

2.4.1 Death process

We modelled the death rates of ages below 85 with the Lee-Carter model [3] and above 85 with the Coale-Kisker method [4].

Aged 0-84 The Lee-Carter model (LCM) is a statistical model decomposing the death rates into an age-specific baseline ($\alpha_{age,sex}$), age-specific trend terms with resect to time effect ($\beta_{age,sex}$), and an intrinsic time trend ($\kappa_{year,sex}$), which is modelled and forecasted using time-series methods. We applied the likelihood-based implementation derived by Brouhns et al. [5] to model the death rates ($d_{year,age,sex}$), where $d_{year,age,sex} = D_{year,age,sex} / PM_{year,age,sex}$. Therefore,

$$log(E(D_{year,age,sex})) = \alpha_{age,sex} + \beta_{age,sex}\kappa_{year,sex} + log(PM_{year,age,sex})$$

We modelled $\kappa_{year,sex}$ using an ARIMA time-series model. Applying the Box–Jenkins method [6], we identified $\kappa_{year,male}$ as a $ARIMA(1,0,0)$ with drift and $\kappa_{year,female}$ as a $ARIMA(0,1,0)$ with drift. We then used the bootstrap method for LCM by Renshaw and Haberman 2008 [7] to generate 10,000 simulations of deaths

Aged 85-100 In the extremely old population, the sample sizes of mortalities might be small which usually leads to high uncertainty in statistical models. The Coale-Kisker method [4] fits exponentially increasing mortalities with age.

Explicitly, our implementation follows the steps: 1) set up a final death rate ($d_{year,100,sex}$) for each sex, 2) rescale $d_{year,84,sex}$ and $d_{year,100,sex}$ to a logarithmic scale, 3) linearly interpolate the $log(d_{year,age,sex})$ for $age \in [85, 99]$, and 4) transform back to the original scale.

We assumed final death rates at age 100 of males and females as 1 and 0.8 as the NDC model to prevent the rates crossing over.

2.4.2 Birth process

As the LCM can can also be applied on fertility rates (e.g. Simpach [8] and Hyndman and Booth [9]), we modelled birth processes using the LCM. Considering the number of newborns and availability of data, we aggregated the population size of females to five-year age-groups (15-19, ... , 45-49). Using the same specification of the method of the death process, we modelled fertility rates as

$$log(E(f_{year,agp,sex})) = \alpha_{agp,sex} + \beta_{agp,sex}\kappa_{year,sex} + log(PM_{year,agp,female})$$

.

For deaths, we then used the bootstrap method to generate the numbers of newborns ($F_{year,agp,sex}$). The total numbers of newborns by sex were

$$B_{year,sex} = \sum_{agp} F_{year,agp,sex}$$

2.4.3 Migration process

We did not use migration data directly, but used the residual method [10] to derive the net migration instead. Under this method, the net migrations were calculated

from the difference between net flows in the population at an age and flows due to birth and death:

$$M_{year,0,sex} = PE_{year,0,sex} - B_{year,sex}$$
$$M_{year,age,sex} = PE_{year,age,sex} - (PF_{year,age,sex} - D_{year,age,sex})$$

Then, the net migration rates are

$$m_{year,age,sex} = M_{year,age,sex} / PM_{year,age,sex}$$

.

In terms of forecasting, we took the arithmetic mean of net migration rate for each single-year age, assuming the future migrations will be constant at a given age. We sampled the number of net migration, $M_{year,age,sex}$, by the Poisson distribution with mean $m_{year,age,sex}$.

2.4.4 Simulation

After the parameter estimation procedures above, we simulated the population dynamics as follows.

Step 1, Initialisation The first step starts with setting up the initial population sizes of each (age, sex). For the population aged above one, the initial size is from the end-year population estimates of the previous year. For the age zero population, the initial size is zero.

Step 2, birth, death and migration For the zero aged populations by sex, add the number of birth to the values. For each single-year age and sex, add the number of net migration and subtract the number of deaths to the population.

Step 3, Ageing Remove the oldest population group ($age = 100$) and count them as deaths. Then, shift the population upward by one year.

Step 4, Iteration Iterate through Step 1 to Step 3 until 2035.

Last, apply the procedure on every bootstrap sample to complete the simulation.

2.5 Notes

Software: The data manipulation, modelling, and forecasting in this document were conducted using R 3.5.1 with packages of StMoMo and TSA [11–13].

Time inconsistency due to ageing process We did not use migration data directly because there is a time inconsistency due to ageing. The time inconsistency of ageing and year progress commonly showed up in the dataset. For example, if a newborn baby who has their birthday in August died in the March of the next year, he/she would be counted in the zero-age death. However, if he/she died in September of the next year, that would be a count in the one age death. The same issue can be found in population and migration data. Therefore, the numbers are always unmatched by age structure. In order to fix this issue, we used the residual method for the migration process, ensuring the other aspects of data were balanced in every year.

Bibliography

[1] National Development Council, Taiwan. Population Projections for the R.O.C. (Taiwan): 2018-2065. National Development Council, Taiwan; 2018.

[2] Alho JM. Stochastic methods in population forecasting. Int J Forecast. 1990 Dec;6(4):521–530.

[3] Lee RD, Carter LR. Modeling and Forecasting U. S. Mortality. J Am Stat Assoc. 1992;87(419):659–671.

[4] Coale AJ, Kisker EE. Defects in data on old-age mortality in the United States: new procedures for calculating mortality schedules and life tables at the highest ages. Coale Kisker 1990 Asian and Pacifc Population Forum. 1990;.

[5] Brouhns N, Denuit M, Vermunt JK. A Poisson log-bilinear regression approach to the construction of projected lifetables. Insur Math Econ. 2002 Dec;31(3):373–393.

[6] Box GEP, Jenkins GM, Reinsel GC, Ljung GM. Time Series Analysis: Forecasting and Control. John Wiley & Sons; 2015.

[7] Renshaw AE, Haberman S. On simulation-based approaches to risk measurement in mortality with specific reference to Poisson Lee–Carter modelling; 2008.

[8] Simpach O. Fertility of Czech Females Could Be Lower than Expected: Trends in Future Development of Age-specific Fertility Rates up to the Year 2050. STATISTIKA-STATISTICS AND ECONOMY JOURNAL. 2015;95(1):19–37.

[9] Hyndman RJ, Booth H. Stochastic population forecasts using functional data models for mortality, fertility and migration. Int J Forecast. 2008 Jul;24(3):323–342.

[10] Hamilton CH. Effect of census errors on the measurement of net migration. Demography. 1966 Jun;3(2):393–415.

[11] R Core Team. R: A Language and Environment for Statistical Computing. Vienna, Austria; 2018.

[12] Andres V, Millossovich P, Vladimir K. StMoMo: Stochastic Mortality Modeling in R. J Stat Softw. 2018;84(3):1–38.

[13] Chan KS, Ripley B. TSA: Time Series Analysis; 2018.
