# Supplementary material for: Forecasting the impact of population ageing on tuberculosis incidence
Source: PLoS One. 2019 Sep 24;14(9):e0222937. doi: 10.1371/journal.pone.0222937 (PMC6759178; doi:10.1371/journal.pone.0222937)
Supplement: S3 Appendix — (PDF) [file pone.0222937.s003.pdf]

Goodness of fit

3.1 Residuals

Figure 3.1: Residuals by age and calender year.

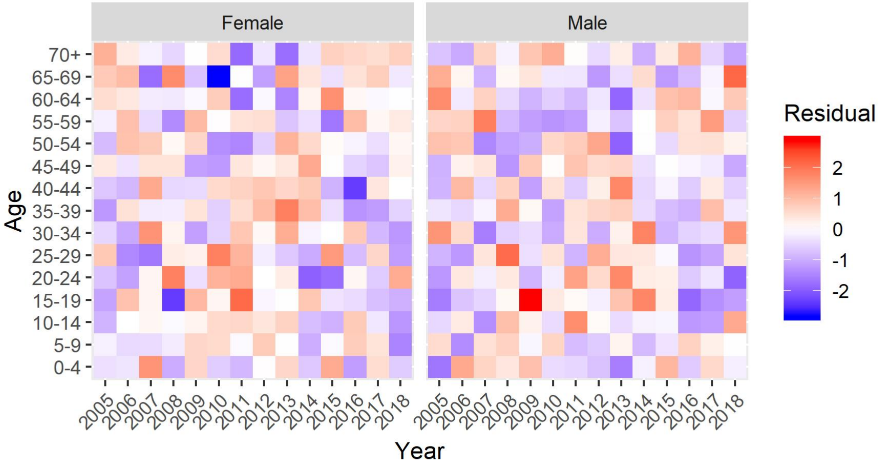

Figure 3.2: Residuals by age and by calender year.

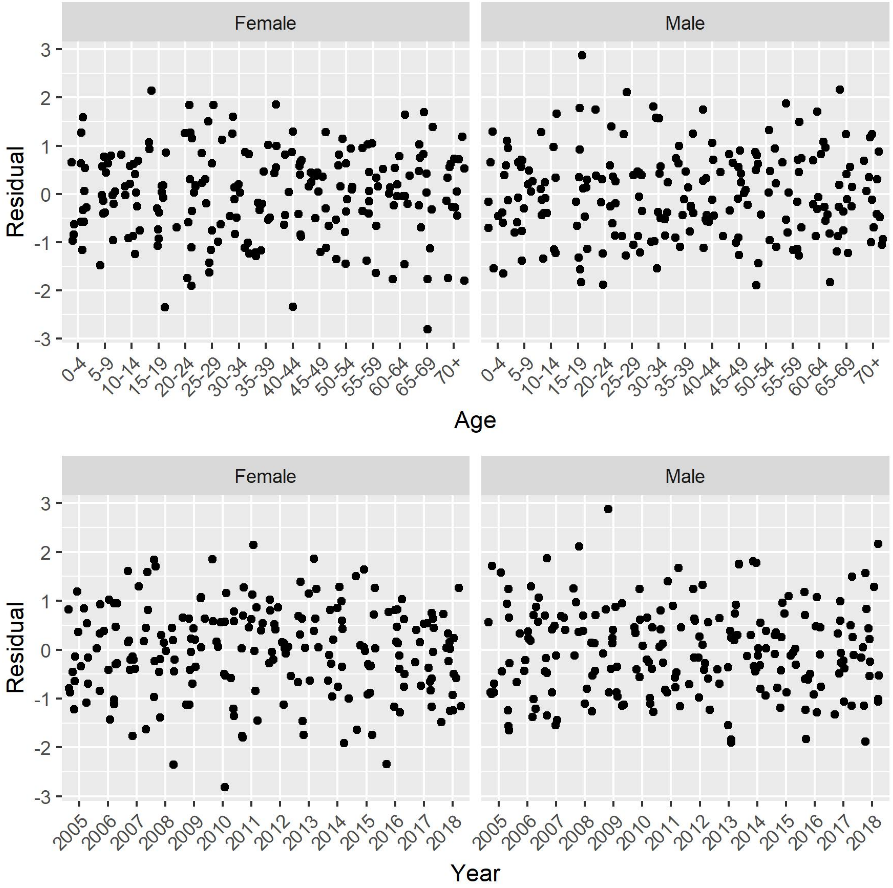

3.2 Fitted TB incidence rates

Figure 3.3: Fitted TB incidence rates in logarithm to the base 10

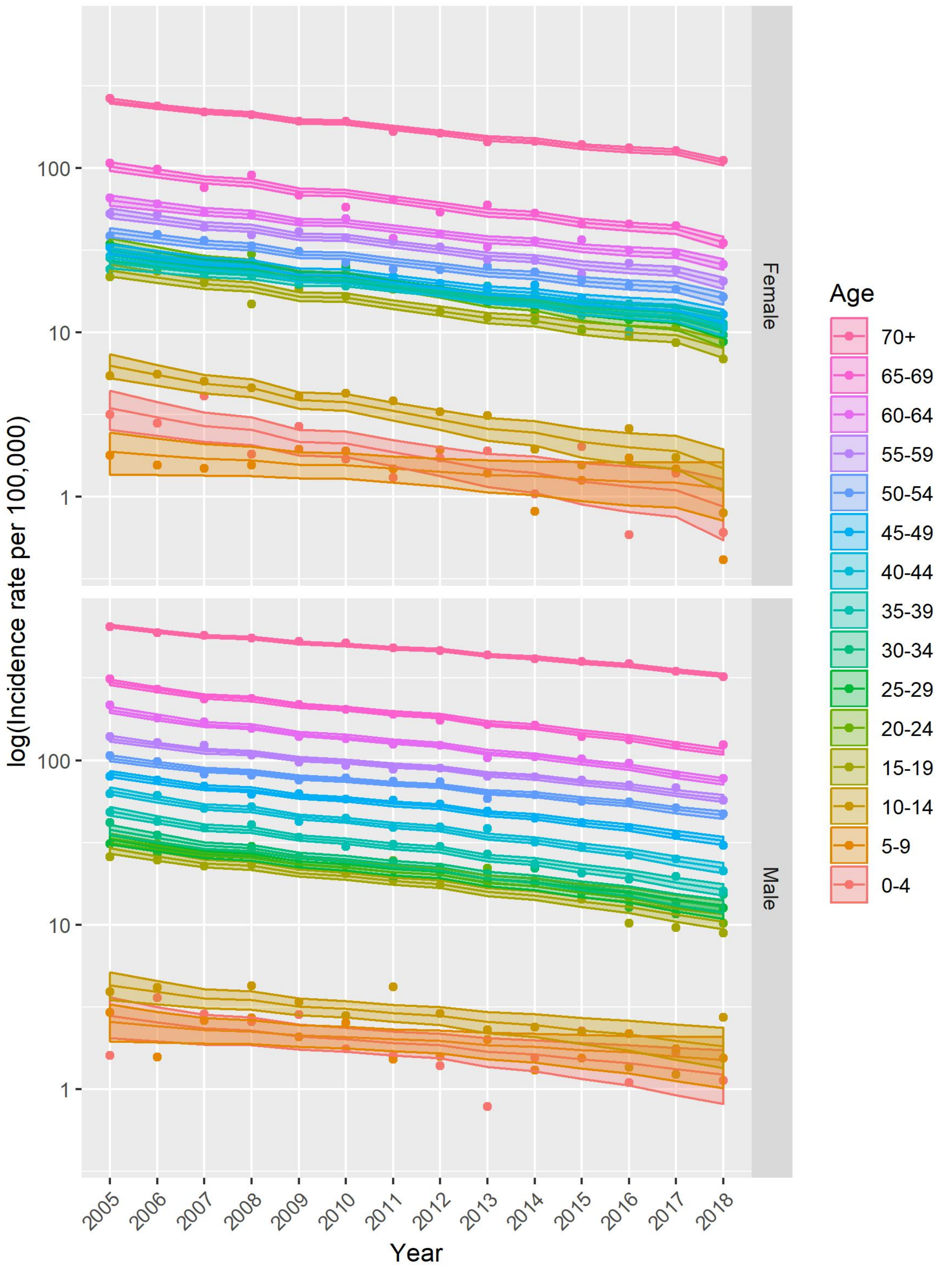

3.3 Validation with disjoint partitions

Training dataset: 2005-2013; validation dataset: 2014-2018

Figure 3.4: Fitted and forecasted TB incidence rates in logarithm to the base 10

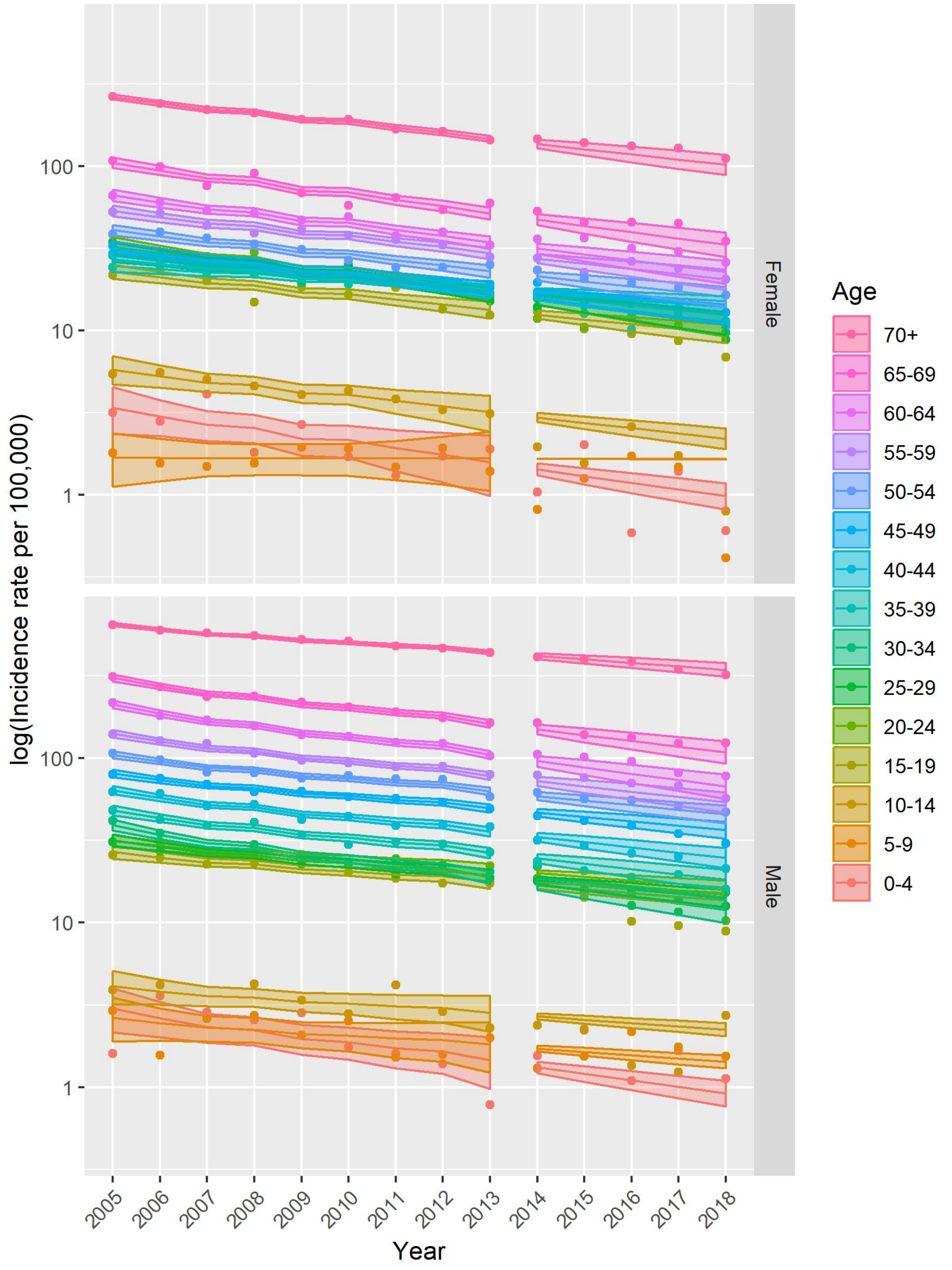

3.4 Model comparison

We modelled female and male TB incidence separately with the same parameterisation. Apart from the likelihood-based Lee-Carter Model, two models were used as comparators. Both models can be seen as reduced Lee-Carter Models:

Age-Trend: A Poisson regression model with categorical age effects and a linear term of the calender year.

Age-Period: A Poisson regression model with categorical age effects and a discretised calender year.

Table 3.1: Model comparison, female

	Age-Trend	Age-Period	Lee Carter Model
Family		Poisson Regression	
Period.effect	Linear	Discrete	
No. observations		210	
No. parameters	16	28	42
Log(Likelihood)	-852	-840	-811
AIC*	1737	1737	1706
BIC†	1790	1830	1847

Table 3.2: Model comparison, male

	Age-Trend	Age-Period	Lee Carter Model
Family		Poisson Regression	
Period.effect	Linear	Discrete	
No. observations		210	
No. parameters	16	28	42
Log(Likelihood)	-1002	-979	-870
AIC	2036	2014	1825
BIC	2090	2108	1965
